# The Effect of Rehabilitation Therapy in Children with Intervened Congenital Heart Disease: A Study Protocol of Randomized Controlled Trial Comparing Hospital and Home-Based Rehabilitation

**DOI:** 10.3390/jcm14030816

**Published:** 2025-01-26

**Authors:** Mónica Menéndez Pardiñas, Ángeles Sara Fuertes Moure, José Manuel Sanz Mengíbar, Fernando Rueda Núñez, Jorge Cabrera Sarmiento, Javier Martín-Vallejo, Rita Jácome Feijoó, Isabel Duque-Salanova, Juan Luis Sánchez González

**Affiliations:** 1 Unidad de Atención Temprana y Rehabilitación Infantil, del Complejo Hospitalario Universitario de A Coruña (CHUAC), 15006 A Coruña, Spain; monicamenendez31@gmail.com (M.M.P.); jorge.jesus.cabrera.sarmiento@sergas.es (J.C.S.); 2 Departamento de Fisioterapia, Medicina y Ciencias Biomédicas de la Universidad de A Coruña (UDC), 15006 A Coruña, Spain; 3 Unidad de Cardiología Infantil, Servicio de Pediatría, Complexo Hospitalario Universitario A Coruña, 15006 A Coruña, Spain; angeles.sara.fuertes.moure@sergas.es (Á.S.F.M.); fernando.rueda.nunez@sergas.es (F.R.N.); 4 Centre for Neuromuscular Diseases, National Hospital for Neurology and Neurosurgery, University College London, London WC1E 6BT, UK; 5 Departament of Stadístics, Faculty of Medicine, University of Salamanca, 37008 Salamanca, Spain; jmv@usal.es; 6 Instituto de Investigación Biomédica de Salamanca (IBSAL), 37007 Salamanca, Spain; juanluissanchez@usal.es; 7 Hospital de día de Pediatría, Complexo Hospitalario Universitario a Coruña, 15006 A Coruña, Spain; rita.jacome.feijoo@sergas.es (R.J.F.); ma.isabel.duque.salanova@sergas.es (I.D.-S.); 8 Faculty of Nursing and Physiotherapy, University of Salamanca, 37008 Salamanca, Spain

**Keywords:** physiotherapy, cardiac rehabilitation, exercise, congenital heart disease, respiratory physiotherapy, exercise capacity, quality of life

## Abstract

**Background/Objectives:** Children who suffer from congenital heart defects (CHDs) have a decreased ability to perform physical exercise and consequently have a decrease in their functional capacity. The main causes of this decrease in functional capacity have been related on the one hand to residual hemodynamic defects and, at the same time, to a situation of physical deconditioning due to inactivity, as well as problems in lung function, especially the presence of restrictive patterns that influence the amount of O_2_ insufflated (decreased maximum VO_2_), consequently generating a deficient maximum O_2_ consumption and maximum work rate. This represents an important prognostic value, since it constitutes an independent predictor of death and hospitalization. This study aims to determine the benefits obtained regarding respiratory function, exercise capacity, and quality of life after implementing a hospital-based cardio-respiratory rehabilitation program compared to a home-based Cardio-respiratory Physical Activity Program in patients with intervened CHDs. **Methods**: This is a randomized controlled trial on the effectiveness of two different rehabilitation programs on respiratory function, exercise capacity, and quality of life in patients with CHDs conducted at the Child Cardiology and Congenital Heart Disease Unit of the University Hospital Complex of A Coruña (CHUAC). There will be two groups: Cardio-respiratory rehabilitation group program conducted in a face-to-face format at the hospital (*n* = 26) and a study group that follows a home-based Cardio-respiratory Physical Activity Program (TELEA) (*n* = 26). The measurement variables will be respiratory function, forced vital capacity (FVC), forced expiratory volume in the first second (FEV1), maximum expiratory flow (PEF), the Tiffeneau index (FEV1 /FVC), forced expiratory flow (FEF25%, FEF50%, FEF75%, FEF25–75%), exercise capacity (peak VO_2_), and the quality of life of these children and their families. **Conclusions**: The implementation of cardiac and pulmonary rehabilitation programs in children with CHDs is essential to improve their quality of life, exercise tolerance, and socialization. These programs optimize life expectancy and promote integration, being crucial for their physical and emotional well-being

## 1. Introduction

Congenital heart defects (CHDs) are structural heart malformations present at birth and represent one of the leading causes of neonatal morbidity and mortality [1]. These anomalies are associated with significant health burdens [2], affecting approximately 1% of newborns. While the precise etiology of CHDs remains largely unknown, approximately 70–80% of cases result from interactions between genetic susceptibility and environmental factors, including maternal health conditions and teratogenic exposures during gestation [3,4].

Advances in medical and surgical management over the past four decades have significantly improved survival rates, with current data indicating that approximately 85% of children with CHDs survive into adulthood, including those with complex defects [5]. The improved survival outcomes, however, do not fully resolve challenges related to quality of life, life expectancy, or comorbidities. Children with CHDs often experience limited exercise capacity due to residual hemodynamic abnormalities and reduced pulmonary function, with a restrictive lung pattern being the most prevalent impairment [6]. Although to a lesser extent, some children may develop obstructive or mixed patterns resulting mainly from surgical manipulation or prolonged use of mechanical ventilation. Lung function deficits, particularly when moderate to severe, are independent predictors of mortality in adult CHD patients [7]. A sedentary lifestyle, frequently imposed on these children due to perceived fragility, exacerbates physical deconditioning and worsens cardiovascular outcomes.

Cardiac rehabilitation programs (CRPs) have demonstrated significant benefits in improving quality of life, exercise capacity, emotional well-being, and self-esteem in CHD patients. These programs also reduce morbidity and mortality in both the short and long term, with minimal reported adverse effects [5,8,9,10,11]. Despite these benefits, the availability and implementation of CRPs for pediatric CHD patients remain insufficient [12]. Pediatric CRPs present unique challenges, requiring family engagement and coordination among multidisciplinary teams experienced in managing CHDs. Effective communication with families is critical for ensuring adherence, particularly in hospital-based programs. While home-based CRPs offer cost advantages for healthcare systems, their effectiveness requires further validation [13,14,15].

Most pediatric CRPs rely on recreational exercise, such as sports (e.g., basketball, dancing, running, or swimming), stretching, and low-resistance weight-bearing activities [11,12,13,14,15,16]. However, restrictive lung dysfunction—a common finding in CHD patients, especially those undergoing cardiothoracic surgery—compounds exercise limitations. Its prevalence increases with younger surgical age and the number of thoracotomies performed [17]. Such pulmonary limitations, including reduced forced vital capacity, are closely linked to diminished exercise capacity, the strongest predictor of survival in this population [18,19].

### Main and Secondary Objectives

Main Objectives:

- To evaluate whether our proposed cardio-pulmonary rehabilitation program improves respiratory function and exercise capacity.
- To compare outcomes between the hospital-based rehabilitation group and the home-based rehabilitation group.

Secondary Objectives:

- To identify whether this specific CRP achieves higher adherence rates compared to other programs reported in the literature.
- To determine if adverse effects occur during or after completing the program.
- To verify whether the program has been conducted in a safe environment and assess its feasibility in natural settings.
- To determine if the program leads to improvements in the quality of life of these children and their families.

## 2. Materials and Methods

A parallel randomized controlled trial with a simple assignment has been designed (1:1) to test the effect of a 12-week specific CRP on respiratory function in pediatric patients with intervened CHDs. The trial will be conducted at the Teresa Herrera Maternal and Child Hospital of the University Hospital Complex of A Coruña (CHUAC) and follow the Consolidated Standards of Reporting Trials (CONSORT) Statement [20]; the treatment protocol is described in accordance with the recommendations of SPIRIT [21].

### 2.1. Study Participants and Setting

Study participation will be voluntary. Each potential participant will receive detailed information regarding the study objectives and methodological procedures prior to providing written informed consent.

#### 2.1.1. Inclusion Criteria

Individual case evaluation will determine eligibility for the Cardiac Rehabilitation Program (CRP). The pediatric cardiologist will assess each case and make decisions in conjunction with the patient’s family and the child or adolescent.

Participant selection will be based on the following criteria:

- Pediatric patients with a history of cardiac transplantation or CHDs who have undergone at least one interventional procedure or surgical intervention, at least 6 months before the start of the program in a state of clinical stability, hemodynamic stability, and ECG stability (ambulatory patient and does not require inotropic support or other therapies). They should have residual hemodynamic defects of sufficient severity that potentially restrict participation in physical activities, and their perception of fragility may be influenced by the social, family, school environment, or by the patient themselves.
- Children aged 8–17 years at the start of the study.
- Present a maximum oxygen consumption figure (peak VO_2_) below 80% of that predicted in ergo-spirometry performed prior to the start of the intervention program (in no case more than 6 months before the start).
- Patients who do not present contraindications for carrying out the program after evaluation in consultation with a pediatric cardiologist and a rehabilitation doctor.

#### 2.1.2. Exclusion Criteria

- Patients with acute, inflammatory, or infectious health conditions that could pose a risk to them during the program.
- Patients who have undergone at least one interventional procedure or surgical intervention in a period of no less than 6 months before the start of the study.
- Lack of cooperation from the child due to immaturity or inability to understand or follow simple instructions required for evaluation or CRP.
- Lack of motivation from the patient or their guardians.
- Patients with comorbidities alongside CHDs that may influence exercise capacity or ventilatory function, or prevent participation in the program, such as acute inflammatory or infectious diseases.
- Withdrawal of informed consent.

### 2.2. Hospital Group

#### 2.2.1. Cardio-pulmonary Rehabilitation Group Program Conducted in Face-to-Face Format at the Hospital

Due to the current absence of standardized exercise physiology protocols in pediatric cardiology [22], we developed a structured program based on cardio-pulmonary physiology and the limited scientific evidence available up to the present moment [13,23,24]. The sessions were group-based (2–3 patients) and supervised by a pediatric physiotherapist, a pediatric nurse, and a pediatrician specialist in cardiology or a pediatric rehabilitation doctor. Patients were encouraged to maintain their habitual activities during the protocol.

The program will take place twice a week for 12 weeks.

Components of the program:

- Respiratory Physiotherapy Program: This part of the program incorporates two distinct device-based respiratory training modalities. Our literature review found no previous documentation of respiratory physiotherapy protocols specific to different CHD classifications [13,23,24,25,26] except for patients with Fontan circulation where only techniques with inspiratory muscle training (IMT) devices [27,28,29] or educational sessions with respiratory awareness techniques [30] were used. The objective is to increase inspiratory volume, since most of the patients with CHDs have a restrictive ventilatory pattern (to be determined after ergo-spirometry analysis) and weakness of the respiratory muscles [31]. Children with obstructive or mixed respiratory patterns will also benefit from this program. Improvements in lung inflation have a direct impact on bronchial obstruction.
  ○ Coach 2 volumetric incentive device with 4000 mL (DHD Healhcare^®^ USA (Coach^®^ 2 Incentive Spirometer, 4000 mL, Smiths Medical ASD, Inc., Plymouth, MN, USA)): The patient maintains an upright seated position with proper trunk alignment, with their feet on the floor and holding the device at eye level with both hands. The physiotherapist will ask the patient to exhale slowly until the RV is reached and immediately inhale slowly to perform a maximum inspiration through the mouthpiece, ensuring lip sealing and trying not to block the mouthpiece with the tongue. The piston must be raised as high as possible. At the same time, the children must ensure that the training indicator on the right, indicative of the inhalation speed, floats between both limits. The highest value of the three procedures performed will be recorded as a reference for the intervention. They will perform three sets of ten repetitions with a 5-s apnea to take advantage of collateral ventilation.
  ○ Threshold IMT (Threshold IMT, Philips^®^ Respironics, Inc., Murrysville, PA, USA): The objective is to enhance inspiratory muscle strength to increase inspiratory volumes. The resistive load creates negative pressure that helps open the airway and allows better air filling in the lung. The participants will be seated with their feet flat on the floor and an upright trunk. It is important to adjust the mouthpiece by closing the lips tightly around it. A nose clip will be worn to ensure that patients are breathing exclusively through the training device; the child must inhale with enough force to overcome the resistance of the spring and for the valve to open the air passage using diaphragm musculature, trying to expand the rib cage, to avoid the use of accessory muscles; we will progressively tighten the device until the pressure in cmH_2_O is reached, until the child can overcome the maximum resistance at which air can enter his lungs (PIM). This procedure will be performed 3 times, and we will take as reference the highest mark. The maneuvers will be supervised the whole time by an experienced physiotherapist. They will perform three sets of ten repetitions at 30% of the maximum tested in the first month. Once the first month is over, the maximum will be calculated again, and work will be performed at 60% of this value. Once the second month is over, the maximum will be calculated again and work will be performed at 60% of the new value.
  ○ Children will take both devices home and perform the same intervention once a day. Duration: 15 min. Each participant kept a diary of usage at home.
- Aerobic training: Before starting the training, nursing staff will measure O_2_ saturation, blood pressure, and heart rate. Continuous aerobic training is the main component of this group. Patients will undergo the training program with the physiotherapist. The child will work within the heart rate ranges established after performing ergo-spirometry. Moderate intensity exercise will be performed. At the beginning of the training, the child will be working with a heart rate closest to the first ventilatory threshold (VT1). Gradually, based on the monitored child’s tolerance and their perception on the Modified Borg scale (0–10), the heart rate will progressively shift towards the second ventilatory threshold (VT2) [32]. Patients will alternate their training, using a Bike ergometer for pediatric use, Ergoselect 150 (Ergoline^®^ GmbH, Bitz, Germany), or a Treadmill RAM 870 clinical (Medisoft^®^ RAM Italia S.r.l., distributed by MGC Diagnostics Corporation, Saint Paul, MN, USA). Training will be conducted under medical supervision with Telemetry monitoring system ers.2 Software ers.2 (Ergoline^®^ REHAB system ers.2 GmbH, Bitz, Germany) for safety, and this will be perceived by the patient and their families. Patients will be taught to identify symptoms and signs to stop physical exercise both in the hospital setting and in natural environments. These symptoms include chest pain, tightness, palpitations, a significant increase in shortness of breath, dizziness, fainting, unusual fatigue and nausea, signs of paleness or cyanosis in the skin, lips, or nails, or cold or sweaty skin. Warm-up exercises = 5 min, continuous aerobic training = 20 min, cool-down exercises = 5 min.
- Resistance training: To perform this part of the training, patients will be monitored at all times to ensure safety. Strength-resistance interval aerobic training will be performed. The lower body will be worked more intensively, including the soleus, gastrocnemius, quadriceps, hamstrings, adductors, and gluteus (Bauer pump). Examples: Wide squat, dynamic lunge, high pitch, butt kicks, high knee pointed toe, etc., along with abdominal and diaphragmatic muscles (thoracoabdominal pump/thoracic suction) and standing percussion exercises to activate the plantar venous pump (Lejars venous sole). Examples: Jump high knee pointed toe, etc. All of these exercises enhance venous return and promote preload of the heart, increasing the efficacy of the Frank–Starling mechanism. In addition, they enhance the expanding respiratory muscles of the thoracic cage, mainly the serratus and pectoralis muscles in the closed kinetic chain. Example: From supine 90/90 hip and knee position to oblique sitting synchronized with breathing. The children will begin with 10 s of work and 10 s of rest, and halfway through the training period, that will increase to 20 s of work and 10 s of rest, while maintaining the proposed heart rate ranges for their training at all times. The program will be increased by introducing the step or low-resistance weights, and calisthenics and avoiding at all times intense isometry or Valsalva. All the proposed exercises will be synchronized with breathing. Duration: 15 min.
- Stretching exercise program: To conclude the program, the physiotherapist will lead stretching exercises, primarily targeting shortened muscles (specific to each patient) and those involved in the training program. Postural-adjusted static and active stretching or active stretching with postural feedback will be performed. Examples: Standing forward fold to hamstring stretch with pelvic lift, prone quad stretch, standing quad stretch with pelvic tilt extension, seated forward stretch with ischial alignment and upright posture, seated wide-leg stretch for adductors and upright posture, seated butterfly stretch and upright posture, dynamic calf and soleus stretch with alternating knee flexion. Duration: 5 min.

Total time for continuous and interval aerobic exercise: 40 min.

Total duration of the complete cardio-pulmonary rehabilitation group: approximately 1 h.

#### 2.2.2. Study Group—Home-Based Cardio-pulmonary Rehabilitation Group

Patients assigned to this group will perform the CRP at their own home. The prescribed physical exercise will be uploaded in video format on the TELEA platform belonging to Galician Health Service (SERGAS). The patients will attend the hospital once at the beginning of the study to learn the program with the physiotherapist, who will tell them what their training heart rate is and the safety ranges in which they should move, and once again at the end of the first month. The program will take place twice a week in their home for 12 weeks. Patients will be encouraged to maintain their habitual activities during the protocol.

**Figure 1:**
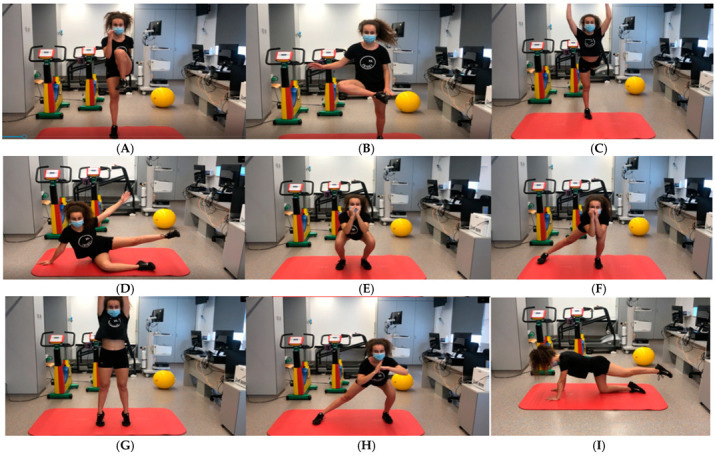
Frames/screenshots from some of the exercises in the TELEA’s video tutorial showing different exercises to strengthen the muscles involved in the venous return pumps visualized through the platform. (**A**) High knee pointed toe, (**B**) standing cross body toe tap, (**C**) standing leg kickback with overhead reach, (**D**) side kick, (**E**) squat, (**F**) lateral sliding lunge, (**G**) standing calf raise with arm lift, (**H**) side lunge with extended leg, (**I**) quadruped leg extension and flexion.

**Figure 2:**
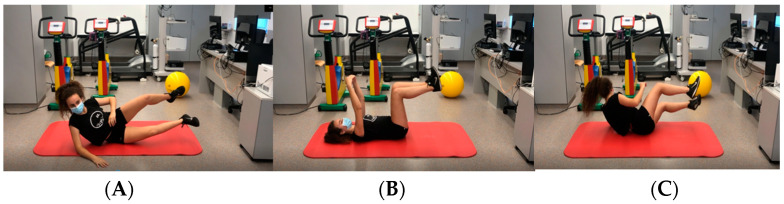
Sequence from supine 90/90 hip and knee position to oblique sitting (following Ontogenic patterns) synchronized with breathing to enhance inspiratory muscles (serratus and pectoralis) in a closed kinetic chain in sync with abdominal muscles. Sequence: (**A**) oblique sitting on right arm, (**B**) supine position, (**C**) oblique sitting on left arm.

Components of the program:

- Respiratory Physiotherapy Program: Children will use two types of respiratory training with devices—COACH and IMT. The home-based rehabilitation program will follow the same respiratory physiotherapy regimen as the hospital-based group, except for the final phase of incremental resistance in IMT. These children will attend the hospital in person only at the start of and at the end of the first month. They will perform three sets of ten repetitions at 30% of the maximum tested in the first month. Once the first month is over, the maximum will be calculated again, and work will be performed at 60% of this value.
  ○ The intervention will be carried out once a day. Duration: 15 min. Each patient will report the completion of the program through the TELEA platform. TELEA is a remote healthcare platform. This platform enables healthcare professionals to monitor and track the health status of patients in their homes, facilitating communication and reducing the need for hospital or clinic visits. Through TELEA, patients can record their vital signs and other health data, which are monitored in real-time by healthcare professionals. TELEA supports communication via videoconferencing and other digital tools, for instance, the video tutorial of the exercise home program, enhancing accessibility and the quality of healthcare services. Children will carry out the home program following the exercises of the video tutorial (Figure 1).
- Aerobic interval training: Patients will be monitored during physical exercise with a Heart Rate Monitor with ANT and Bluetooth wireless technology (HRM-Dual™), Garmin Ltd., Olathe, KS, USA. Legally domiciled in Schaffhausen, Switzerland) and GPS running watch Forerunner 45S (GARMIN^®^ Ltd., Olathe, KS, USA. Legally domiciled in Schaffhausen, Switzerland). The child will work within the heart rate ranges established after performing ergo-spirometry. Moderate intensity exercise closest to the first ventilatory threshold (VT1) will be performed. Gradually, based on the monitored child’s tolerance and their perception on the Modified Borg scale (0–10), the heart rate will progressively shift towards the second ventilatory threshold (VT2). They and their families will be taught to identify the symptoms and signs to stop physical exercise, just like the control group. Patients will download their heart rate and Modified Borg scale data after each session and can establish contact through the TELEA platform with the nursing staff of the cardiac rehabilitation unit at all times to resolve any questions they may have. Children in this group will not perform continuous aerobic exercise. They will perform interval aerobic exercise through bodyweight-based strength endurance training, following the same physiological principles explained for the control group. Exercises like wide squat, dynamic lunge, high pitch, jump, butt kicks, etc., will be performed. Some of them are shown in Figure 1 and Figure 2. Duration: 40 min.
- Stretching Exercise Program and Cool-down Exercises: to conclude the program, stretching exercises will be performed just like the control group. Duration: 5–10 min.
  Total duration of the complete program: approximately 1 h.
  (Figure 1 and Figure 2).

### 2.3. Variable and Outcomes: Type and Measurement (Table 1)

The pediatric nurse will conduct a personal interview to collect the following data:

- Demographic information such as name and surname, age and sex.
- Anthropometric measurements: height, weight, circumference of body, and composition.
- Relevant family history and associated comorbidity.
- Personal clinical information related to the condition: diagnoses (especially previous episodes of asthma or bronchospasm), surgical interventions, complications, functional alterations, medication, and timings.
- Sports habits during and outside school: type of sport practiced, and the amount of time spent per week.
- Rehabilitation diagnosis: a pediatric rehabilitation specialist will perform a comprehensive examination to identify any contraindications or biopsychosocial factors that may affect the implementation of the CRP.
- Quality of life questionnaires for children and adolescents with heart disease—PedsQL™ 4.0 Generic Core Scales and PedsQL™ Cardiac Module 3.0. Both questionnaires will be administered to the child, mother, and father separately.

**Table 1:** Evaluation flowchart.

Timeline	Evaluation Flowchart
First Visit	Ergo-spirometry Selection of patients. Provide written and verbal information to the patient and their family regarding the study and the procedures to be conducted. Obtaining informed consent.
Second Visit	Personal interview and measurement of vital signs. Quality of life questionnaires for children and adolescents with heart disease—PedsQL™4.0 Generic Core Scales and PedsQL™ Cardiac Module 3.0.

#### Outcomes (Table 2)

- Assessment of respiratory function/spirometry: Spirometry is the study of choice in pulmonary function laboratories. The parameters it measures are the volume of air that the child breathes in or out in absolute value or related to time; and it is shown in the volume/time curve [33]. It also measures the flow expressed in curves with the flow/volume ratio. The mediations can be simple if slow breathing maneuvers not dependent on time are used or forced if maximum effort is requested in the shortest possible time, thus allowing the study of dynamic volumes and forced flows. The recording will be on a flow/volume curve. The most useful functional parameters obtained after spirometry are forced vital capacity (FVC), forced expiratory volume in the first second (FEV1), maximum expiratory flow (PEF), and the Tiffeneau index FEV1/FVC, FEF25%, FEF50%, FEF75%, FEF25–75%. [34]. The recording is called a spirogram.
- Classify, based on the results of spirometry, whether the child has restrictive [35], obstructive, or mixed ventilatory pattern:
- Restrictive pattern: defined as FVC and FEV1 < 80% of the predicted value, with FEV1/FVC > 80% or normal.
- Obstructive pattern: defined as FEV1/FVC ratio < 70%, FEV < 80%, and normal FVC.
- Mixed pattern: defined as FEV1 < 80%, FVC < 80%, and FEV1/FVC ratio that may be normal, increased, or decreased.
- Exercise capacity: Pediatric treadmill ergo-spirometry will be used. This test allows a non-invasive evaluation of the child’s functional capacity by calculating maximum oxygen consumption. It will be performed in our hospital on a treadmill following the Bruce protocol [36]. The Bruce protocol is a diagnostic tool increasingly used in the outpatient follow-up of these patients, in order to obtain an objective evaluation of the exercise capacity of children with CHDs and to know their real physical situation. It also has undeniable value for prescribing an individualized rehabilitation and physical exercise program. Exercise intensity is determined based on heart rate. This will be determined within the transition zone between the first ventilatory threshold (VT1) and the second ventilatory threshold (VT2). The values obtained in ergo-spirometry performed 6 months prior to the start of the intervention program will be used. Within two weeks after completing the cardio-respiratory physiotherapy program, a new test will be performed for comparative analysis.
- Quality of life: quality of life questionnaires for children and adolescents with heart disease—PedsQL^™^ 4.0 Generic Core Scales and PedsQL™ Cardiac Module 3.0.

**Table 2:** Evaluation protocol.

Measurements	Variable	Material and Methods	Unit of Measurements	Measurements	
				Pre	Post
Lack of adherence to the protocol	Absence	Absence in more than 20% of the sessions	Absence equivalent to missing 5 out of the 24 planned sessions		
Anthropometric data	Circumference of body	Flexible tape measure	cm	X	X
	Composition	Bioelectrical impedance	% body fat	X	X
	Height	Height meter	m/cm	X	X
	Weight	Bascule	kg	X	X
Ergo-spirometry	Oxygen consumption	Ergometer and Treadmill	VO_2_	X	X
Respiratory function	FEV1, FVC, FEV1/CVF, PEF, FEF25%, FEF50%, FEF75%, FEF25–75%	Spirometry	%	X	X
	Respiratory Pattern		Restrictive, Obstructive, and mixed	X	X
Perceived effort	Effort	Modified Borg scale	0–10	X	X

Note: m = meter; cm = centimeter; kg = kilogram; FEV1 = Forced expiratory volume in 1 s; FVC = Forced vital capacity; FEV1/CVF = Tiffeneau index; PEF = maximum expiratory flow; FEF = forced expiratory flow.

### 2.4. Sample Size

Since no information has been found on the magnitude of the effect on the topic studied, an effect size of 0.8 has been set, taking into account the scale proposed by Cohen and meta-analysis on the effect of rehabilitation in other respiratory conditions [37]. Sample size calculation was performed for a mean difference analysis of independent groups. The effect size was d = 0.8 alpha level of 0.05, power 80%. The estimated sample size was 26 subjects per group. Considering losses of 10%, the number of subjects needed was 56 subjects. G*Power 3.1 software was used to calculate the sample size.

### 2.5. Randomization

Participants who meet the inclusion/exclusion criteria and wish to participate in the study will be randomly assigned to either the face-to-face treatment group or TELEA control group. We shall carry out a permutation block randomization (1:1) by generating a set of random numbers where the even numbers define a block of specified ordered random treatment assignments and the odd numbers define the other order of assignment of treatments. The random permuted blocks method should achieve balanced experimental groups. All children from outside the municipality’s constituency (from both groups) will be offered financial help to travel to the hospital.

### 2.6. Data Analysis

The normality of the data distribution was analyzed with the test of Kolmogorov–Smirnov. For the descriptive analysis, the mean and standard deviation were used if the distribution of the variables was normal; otherwise, the median and interquartile range were calculated. The qualitative variables of the study will be defined by frequencies and percentages.

Student’s *t* test will be used to compare the intergroup means if the distribution of the variables is normal. The non-parametric Mann–Whitney U test will be applied if the normality of the data is not fulfilled. Levene’s test will be used to test the equality of variances between groups. Welch’s *t*-test will be used if the assumption is not met. Repeated measures t-test or the non-parametric Wilcoxon t-test will be applied for the comparison of intragroup means depending on the normality of the data. The Chi-Square test will be used to test the association between categorical variables.

A confidence level of 95% will be considered for the calculation of the confidence intervals and a significance level of 5% for the statistical tests. The statistical program IBM SPSS Statistics version 28.0.1 will be used to analyze the data.

## 3. Discussion

There is growing evidence that postoperative patients with CHD should engage in physical exercise training. However, CRPs in pediatric populations remain significantly underutilized, and clinical research into this promising form of intervention is notably sparse. Currently, there is no standardized framework for designing such programs or a clear understanding of their outcomes; only a limited number of clinical trials have investigated the optimal exercise training regimens or strategies to improve adherence in postoperative CHD patients. It is essential to explore exercise modalities that optimize training effects and enhance the quality of life of these patients [38].

The pediatric CHD population has unique and complex needs, suggesting the potential value of exploring innovative intervention beyond general physical exercise. These may include strategies such as ventilatory function recovery, specific strength training based on neurophysiological principles, and comparative analyses of continuous versus interval aerobic exercise. Further research is needed to tailor interventions to the specific characteristics of this patient group [39].

Interval-based aerobic exercise has proven effective in improving functional capacity in cardiovascular patients. This training modality significantly increases VO_2_ max, reflecting enhanced cardiovascular and pulmonary efficiency and improving exercise tolerance. These benefits are particularly relevant in patients with cardiovascular conditions, as they optimize oxygen consumption, exercise capacity, and overall quality of life. Although the superiority of continuous aerobic interval exercise has been established in adults with cardiovascular diseases [40], there are few studies incorporating interval exercise into CRPs for pediatric CHD patients [29]. The CRP proposed in this study integrates both continuous and interval aerobic exercise, which could serve as a model for future research.

The success of CRPs relies on the collaboration of multidisciplinary healthcare teams with specialized expertise and addressing the high associated costs. Adherence challenges are particularly prevalent in hospital-based CRPs, which is why home-based programs are increasingly favored. These home interventions not only reduce costs but also diminish the burden on families. The challenge remains to demonstrate that the benefits in quality of life and functional outcomes are at least equivalent to those achieved in clinical settings [13,14,15].

Incorporating respiratory physiotherapy into CRPs is logical, given that CHD patients often experience impaired pulmonary function, contributing significantly to morbidity and mortality. These impairments are frequently due to restrictive respiratory patterns resulting from thoracotomies, sternotomies, and ventilatory muscle weakness, which collectively worsen lung function [41]. Exploring the potential of specific respiratory physiotherapy techniques, such as volumetric incentive spirometry and IMT, in conjunction with physical exercise, could strengthen respiratory muscles and address cardio-respiratory dysfunction in this population. Although preliminary studies have begun to evaluate the effects of IMT on lung and exercise capacity in pediatric CHD patients [28,42], research remains limited. We propose expanding CRPs to include targeted interventions for enhancing inspiratory volume, and strengthening ventilatory muscles may have broader implications for improving the health status of pediatric CHD patients. Given the scarcity of research in this area, further investigations are required to establish a foundation for optimizing rehabilitation strategies.

It has been documented that during physical exercise, the muscle groups requiring the highest oxygen demand are the respiratory and locomotor muscles, particularly the serratus anterior and the vastus lateralis of the quadriceps. However, these muscles have not been sufficiently targeted in previous CRP studies [13,16,24,26]. Although diaphragm dysfunction has been identified in CHD patients and is linked to restrictive ventilation disorders and exercise intolerance [43], only a few reports have explored the effects of respiratory interventions in patients with a Fontan circulation [27,28,29,30,42]. To date, no controlled clinical trial has examined the full scope of an exercise training program integrated with a comprehensive respiratory physiotherapy regimen in children with CHDs. This highlights the pressing need to describe and implement a structured CRP based on neurophysiological mechanisms.

The program proposed in this study focuses on identifying the specific muscles that require targeted training, distinguishing it from other published programs. Existing programs often advocate general strengthening exercises, such as using wall pulleys and hand weights, without specifying muscle groups or their selection rationale, or rely on general sports activities or games [44]. We propose emphasizing muscles that improve lung inflation (e.g., serratus anterior, pectoralis major) and enhance cardiac preload, as well as lower extremity muscles that act as venous return pumps; for example, the Bauer system—a network of deep veins in the legs surrounded by muscles that facilitate blood return to the heart during contraction and relaxation—and Lejar’s soleus veins, which are critical for venous return during walking [45]. This contrasts with the focus on upper extremity strengthening, which requires higher ventilatory effort compared to equivalent work performed by the lower limbs [46].

### 3.1. Limitations

The results are subject above all to the type of family from which the patients come, influencing the economic level. At this point, and so that this bias is minimized, we propose in our program to provide families with financial transportation assistance in case of belonging to the in-person group.

Children with CHDs are accompanied by high comorbidity. Changes in the process could mean the loss of some subjects from the study. However, we consider that this limitation is the norm in any complex clinical process such as the one at hand.

Children and adolescents suffering from CHDs have been subjected to numerous surgical interventions, medical consultations, and check-ups that have caused multiple absences in their school life. This could cause a lack of adherence to treatment or possible dropouts, especially during the examination stages.

### 3.2. Ethics and Dissemination

This study is being conducted in compliance with the protocol and the principles outlined in the current version of the Declaration of Helsinki [47]. Approval for this study was obtained from the Committee on Clinical Research Ethics of Galicia (CAEI) (registration number: 2017/567).

All participants will be fully informed about this study’s purpose and will provide written informed consent prior to participation. Participant privacy will be safeguarded, with adherence to applicable data protection regulations, and all published data will maintain participant anonymity. Medical information collected during this study will be treated as confidential and will not be shared with third parties. To ensure privacy, each participant will be assigned a randomly generated identification number. Treatment plans will be personalized, with adjustments made as needed to prioritize patient safety and deliver the most tailored care possible. The risk of therapy-related adverse events is considered minimal.

In the event of serious adverse events during or after this study, the participants will be closely monitored by the study team and physicians in line with standard care protocols. All serious adverse events will be documented and reported to the study’s principal investigator within 24 h.

This study will uphold participants’ rights and protect the confidentiality of their data. Each participant will be assigned a unique trial identification number. Personal information will be securely stored at individual study sites in accordance with the 1988 Data Protection Act and will not be included in the trial database. Personal data will only be retained as long as necessary for the purposes for which it was collected.

## Data Availability

The original contributions presented in the study are included in the article, further inquiries can be directed to the corresponding authors.
